# Evaluating the Potential Infectious Risk Profile of Biologics in Chronic Rhinosinusitis With Nasal Polyposis

**DOI:** 10.1002/alr.70201

**Published:** 2026-06-09

**Authors:** Maxime Fieux, Philippe Gevaert, Leigh J. Sowerby

**Affiliations:** ^1^ Centre Hospitalier Universitaire De Lyon Sud, Service ORL et chirurgie cervico‐faciale Pierre Bénite France; ^2^ Université De Lyon, Université Lyon 1 Lyon France; ^3^ UMR 5305, Laboratoire de Biologie Tissulaire et D'ingénierie Thérapeutique, Institut de Biologie et Chimie des Protéines, CNRS/Université Claude Bernard Lyon 1, 7 Passage du Vercors, CEDEX Lyon France; ^4^ Upper Airways Research Laboratory, Department of Head and Skin Ghent University Ghent Belgium; ^5^ Department of Otolaryngology University of Western Ontario London Ontario Canada

**Keywords:** adverse events, biologics, infection, tezepelumab, TSLP

1

1 **Key Points**

- Biologic therapy has been transformative for treating severe and recalcitrant chronic rhinosinusitis with polyposis by targeting different parts of the type 2 inflammatory cascade; downstream effects in immune function may be possible
- TSLP is an epithelial‐derived alarmin that drives type‐2 inflammation but also supports antiviral CD8+ T‐cell responses—blockade which could theoretically impair local antiviral immunity.
- Pooled data from WAYPOINT reveals an increased odds ratio (2.76) for infectious adverse events. This is different from all other CRSwNP biologics trials. Continued vigilance, long‐term surveillance, and guidance on vaccination is warranted.

Biologic therapy for chronic rhinosinusitis with nasal polyposis (CRSwNP) targets specific parts of the type 2 inflammatory cascade and has been transformative for disease control. There is a possibility of unexpected downstream effects of blockade with immunosuppression, but thus far, this has not borne out to be a clinical concern. Thymic stromal lymphopoietin (TSLP) is the newest target for therapy with monoclonal therapy and has a broader role in immune function than previous targets. It is an interleukin (IL)‐2 family alarmin released by human respiratory epithelial cells in response to epithelial injury. TSLP expression is upregulated by epithelial cells, with peak levels occurring early after pathogen exposure. Once released, TSLP binds to a heterodimeric receptor composed of TSLP‐R and IL‐7Rα, initiating a type 2 polarization of naïve T cells and promoting the secretion of cytokines such as IL‐4, IL‐5, and IL‐13. TSLP plays a critical role in epithelial barrier integrity and the orchestration of innate and adaptive immune responses. Its inhibition may theoretically impair mucosal immunity, potentially increasing susceptibility to viral respiratory infections. Two isoforms of TSLP exist in humans, a long and a short form, displaying distinct immunological functions. TSLP is also a critical factor in the maintenance and expansion of Th2‐multipotent progenitor cells [1]. These progenitors exhibit self‐renewal capacity and the potential to differentiate into a spectrum of effector Th2 subsets. TSLP promotes their survival and confers resistance to glucocorticoid‐induced apoptosis, potentially explaining the persistence of inflammation despite steroid therapy in CRSwNP. However, TSLP also supports local proliferation and survival of antiviral CD8+ T cells in the respiratory tract, and its absence or blockade can delay viral clearance and increase morbidity in preclinical models [2].

Tezepelumab, a monoclonal antibody targeting TSLP, has emerged as a promising therapeutic option for patients with severe asthma with activity further up the type 2 inflammatory pathway [3]. Its broad efficacy across eosinophilic and non‐eosinophilic asthma phenotypes has positioned it as a valuable addition to the biologic landscape for pulmonologists. Recently, a phase III randomized and placebo controlled clinical trial (RCT) entitled WAYPOINT demonstrated similar efficacy for the management of CRSwNP with tezepelumab [4].

Given the above information, we assessed the risk of infection in the five landmark RCTs for biologics in CRSwNP, including WAYPOINT. We also assessed tezepelumab‐treated patients from RCTs assessing efficacy in asthma patients.

In CRSwNP, the WAYPOINT trial comparing tezepelumab to placebo has demonstrated efficacy in reducing nasal polyp burden and improving symptom scores [4]. The infectious adverse events (AEs) reported in OSTRO, SYNAPSE, POLYP 1/2, SINUS‐52, and ANCHOR 1/2 were not superior in the treatment group versus the control groups (Table 1). In WAYPOINT, these events were not statistically significant when taken in isolation (Table 2). However, when the reported infectious AEs in WAYPOINT are pooled together, there is an odds ratio of 2.76 (95% CI 1.84, 4.13; (*p* = 0.0000102) for an infectious AE. Odds ratio of total infectious‐related AEs per biologics are available in Figure 1. Of note, the AE of “CRSwNP exacerbations,” which could be treated with either systemic corticosteroids or antibiotics, were excluded due to the ambiguity of infectious or inflammatory etiology. While current data do not demonstrate a statistically significant increase in individual or serious infections, the immunological implications of TSLP blockade warrant further investigation, particularly in patients with comorbidities or those receiving concurrent immunosuppressive therapies. It is also important to consider that WAYPOINT was conducted during the COVID‐19 pandemic, a unique timeline, and some of the signals seen (higher incidence of Upper Respiratory Infection (URI)/nasopharyngitis) may well be secondary to societal normalization and may not be seen again.

**FIGURE 1:**
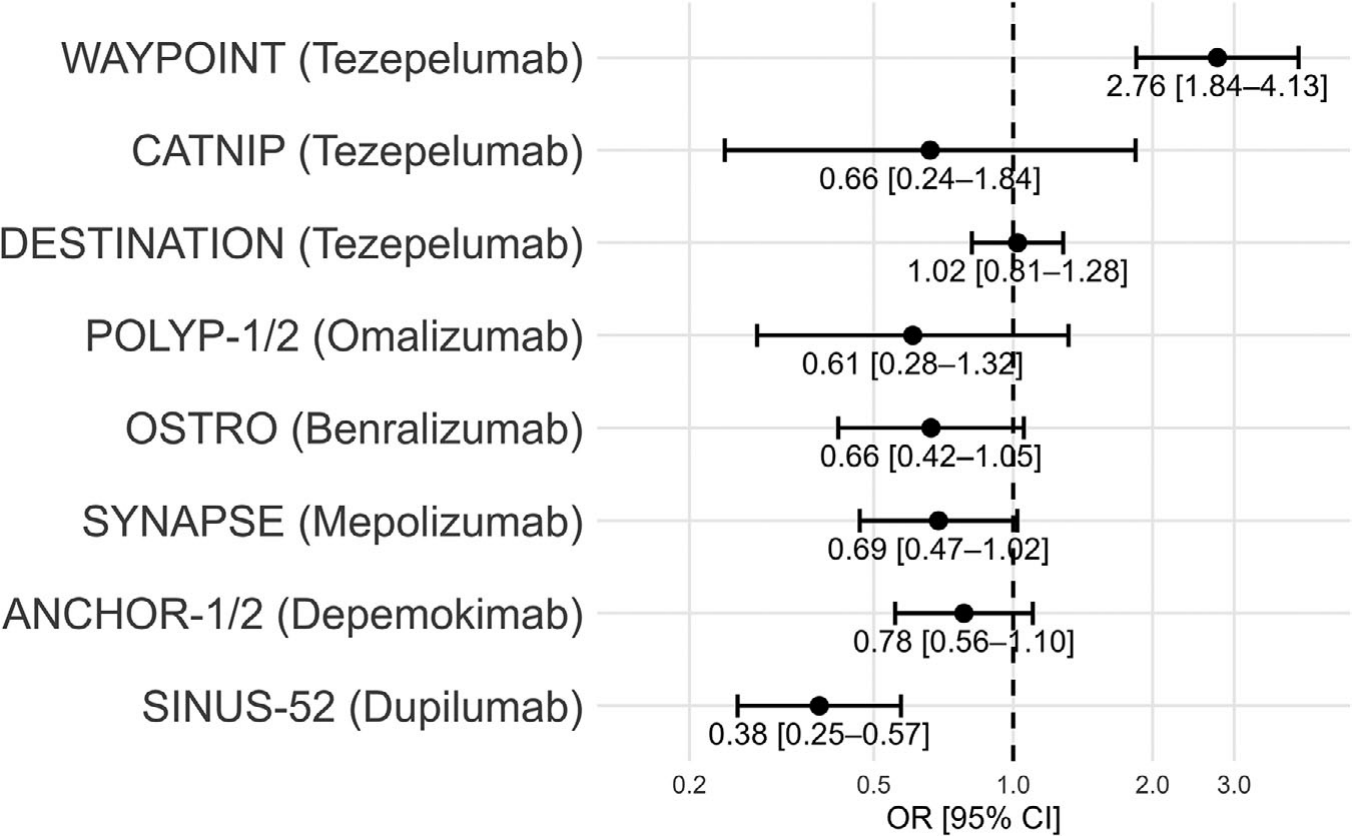
Odds ratio of total infectious‐related adverse events per biologics. *Note*: Odds ratio with 95% confidence interval based on total infectious‐related adverse events available in the original studies.

**TABLE 1:** Adverse events infections in randomized controlled trial assessing biologics.

		Active arm (biologic)				Placebo	OR 95% CI
Trial (biologic)	Population	Nasopharyngitis, *n* (%)	URI, *n* (%)	Others, *n* (%)	Total infectious AE, *n* (%)	Total infectious AE *n*(%)	Total infectious AE
WAYPOINT [5] (tezepelumab)	CRSwNP	36 (17.7)	19 (9.4)	72 (35.5)	137/203 (67.5)	88/205 (42.9)	2.76 (1.84–4.13)
CATNIP [6] (tezepelumab)	Allergic rhinitis	9 (28.1)	6 (18.8)	2 (0.1)	15/32 (53.1)	16/28 (57.1)	0.66 (0.24–1.84)
DESTINATION [7] (tezepelumab)	Severe asthma	146 (24.2)	83 (13.7)	38 (6.3)	267/602 (44.4)	266/607 (42.1)	1.02 (0.81–1.28)
POLYP‐1/2 [8] (omalizumab)	CRSwNP	8 (5.9)	0 (0)	7 (5.2)	12/135 (11.1)	18/130 (13.8)	0.61 (0.28–1.32)
OSTRO [9] (benralizumab)	CRSwNP	36 (17.4)	5 (2.4)	0 (0)	41/207 (19.8)	55/203 (27.1)	0.66 (0.42–1.05)
SYNAPSE [10] (mepolizumab)	CRSwNP	52 (25)	12 (6)	38 (18.4)	102/206 (49.5)	118/201 (58.7)	0.69 (0.47–1.02)
ANCHOR‐1/2 [11] (depemokimab)	CRSwNP	49 (18)	28 (10)	71 (26.1)	130/272 (47.8)	138/256 (53.9)	0.78 (0.56–1.10)
SINUS‐52w [12] (dupilumab)	CRSwNP	61 (20.5)	18 (6.0)	41 (13.8)	120/297 (40.4)	96/150 (64.0)	0.38 (0.25–0.57)

*Note*: Total infectious adverse events included: nasopharyngitis, URI, and others (sinusitis, pharyngitis, influenzae, COVID‐19, otitis media) depending on items reported in the study. Dupilumab (SINUS), mepolizumab (SYNAPSE), depemokimab (ANCHOR‐1/2), and tezepelumab (WAYPOINT) show higher rates of total infectious‐related adverse events. Benralizumab (OSTRO) and omalizumab (POLYP‐1/2) have lower rates of total infectious‐related adverse events.

Abbreviations: AE, adverse event; CRSwNP, chronic rhinosinusitis with nasal polyps; URI, upper respiratory infection.

**TABLE 2:** Summary of infectious adverse events in the WAYPOINT trial.

Adverse events	Tezepelumab	Placebo
Serious infectious adverse events	5	4
Appendicitis	1	0
COVID‐19 pneumonia	1	0
Escherichia urinary tract infection	1	0
Pneumonia bacterial	1	0
Pulmonary tuberculosis	1	0
Bacterial sepsis	0	1
Encephalitis	0	1
Otitis media	0	1
Pneumonia	0	1
		
Mild or moderate adverse events		
Pharyngitis	9	1
Influenza	8	2
Viral upper respiratory tract infection	8	6
COVID‐19	47	40
Nasopharyngitis	36	20
Upper respiratory tract infection	19	11
Total adverse events	137	88

*Note*: Numbers correspond to the number of patients for whom infectious adverse events were reported in the WAYPOINT trial.

In asthma, long‐term safety data from the DESTINATION trial (a phase 3 randomized controlled trial built as an extension of the NAVIGATOR and the SOURCE trial) and phase IV real‐world evidence from the PASSAGE study have not revealed new infectious safety signals. Indeed, when pooling infectious AE from NAVIGATOR AND SOURCE, there were 38.0% cases of nasopharyngitis and/or upper respiratory tract infection in the tezepelumab group and 39.7% in the placebo group. In the PASSAGE study, infectious AEs were reported as 3.8% (8/208 patients). However, authors underscore the importance of continued monitoring in ongoing and future studies. DESTINATION included guidance on COVID‐19 vaccination timing, reflecting concerns about potential immunomodulatory effects of TSLP inhibition [13]. Additionally, pharmacovigilance analyses of the FDA Adverse Event Reporting System (FAERS) identified over 2000 AE reports associated with tezepelumab. The median time to onset of these events was 35 days, suggesting a possible temporal relationship with immune modulation, but no infectious signal was seen [13, 14]. The inherent weakness and bias, however, with FAERS studies are voluntary reporting of side effects.

Considering these findings, we advocate for heightened clinical vigilance regarding infectious risk in patients receiving tezepelumab and reporting of such events to surveillance databases. At this point, it is uncertain whether there is a potential risk of increased bacterial infection given the mechanism of action of TSLP. Indeed, there is a safety signal within WAYPOINT, but the other tezepelumab studies (DESTINATION, PASSAGE) were reassuring. Long‐term real‐world data and post‐marketing surveillance will be critical in fully elucidating the infectious risk profile of this novel biologic therapy. Clear guidance on vaccination timing, infection monitoring, and risk stratification will also be welcomed.

## Funding

MF has received lecture fees and/or participation at expert board meetings from Sanofi, GlaxoSmithKline, and AstraZeneca. LJS has received lecture fees from Sanofi, AstraZeneca, and GlaxoSmithKline and gives trial support to GSK, AstraZeneca, Sanofi, Eli Lily, and Insmed. PG has received lecture fees and/or participation at expert board meetings from Amgen, Argenx, AstraZeneca, Eli Lilly, GlaxoSmithKline, Insmed, Novartis, Regeneron, Roche, Sanofi, and Stallergenes Greer.

## Conflicts of Interest

The authors declare no conflicts of interest.

## References

[1] R. Kratchmarov , S. Djeddi , G. Dunlap , et al., “TCF1‐LEF1 Co‐Expression Identifies a Multipotent Progenitor Cell (TH2‐MPP) Across Human Allergic Diseases,” Nature Immunology 25, no. 5 (2024): 902–915, 10.1038/s41590-024-01803-2. 38589618 PMC11849131

[2] R. Ebina‐Shibuya , E. E. West , R. Spolski , et al., “Thymic Stromal Lymphopoietin Limits Primary and Recall CD8+ T‐Cell Anti‐Viral Responses,” eLife 10 (2021): e61912, 10.7554/eLife.61912. 33439121 PMC7806261

[3] T. M. Laidlaw , A. Menzies‐Gow , S. Caveney , et al., “Tezepelumab Efficacy in Patients With Severe, Uncontrolled Asthma With Comorbid Nasal Polyps in NAVIGATOR,” Journal of Asthma and Allergy 16 (2023): 915–932, 10.2147/JAA.S413064. 37692126 PMC10488831

[4] B. J. Lipworth , J. K. Han , M. Desrosiers , et al., “Tezepelumab in Adults With Severe Chronic Rhinosinusitis With Nasal Polyps,” New England Journal of Medicine 392, no. 12 (2025): 1178–1188, 10.1056/NEJMoa2414482. 40106374

[5] B. J. Lipworth , J. K. Han , M. Desrosiers , et al., “Tezepelumab in Adults With Severe Chronic Rhinosinusitis With Nasal Polyps,” New England Journal of Medicine 392, no. 12 (2025): 1178–1188. 40106374 10.1056/NEJMoa2414482

[6] J. Corren , D. Larson , M. C. Altman , et al., “Effects of Combination Treatment With Tezepelumab and Allergen Immunotherapy on Nasal Responses to Allergen: A Randomized Controlled Trial,” Journal of Allergy and Clinical Immunology 151, no. 1 (2023): 192–201. 36223848 10.1016/j.jaci.2022.08.029 PMC12205947

[7] A. Menzies‐Gow , M. E. Wechsler , et al., “Long‐Term Safety and Efficacy of Tezepelumab in People With Severe, Uncontrolled Asthma (DESTINATION): A Randomized, Placebo‐Controlled Extension Study,” Lancet Respiratory Medicine 11, no. 5 (2023): 425–438. 36702146 10.1016/S2213-2600(22)00492-1

[8] P. Gevaert , T. A. Omachi , J. Corren , et al., “Efficacy and Safety of Omalizumab in Nasal Polyposis: 2 Randomized Phase 3 Trials,” Journal of Allergy and Clinical Immunology 146, no. 3 (2020): 595–605. 32524991 10.1016/j.jaci.2020.05.032

[9] C. Bachert , J. K. Han , M. Y. Desrosiers , et al., “Efficacy and Safety of Benralizumab in CRSwNP: A Randomized, Placebo‐Controlled Trial,” Journal of Allergy and Clinical Immunology 149, no. 4 (2022): 1309–1317 e12. 34599979 10.1016/j.jaci.2021.08.030

[10] J. K. Han , C. Bachert , W. Fokkens , et al., “Mepolizumab for CRSwNP (SYNAPSE): A Randomised, Double‐Blind, Placebo‐Controlled, Phase 3 Trial,” Lancet Respiratory Medicine 9, no. 10 (2021): 1141–1153. 33872587 10.1016/S2213-2600(21)00097-7

[11] P. Gevaert , M. Desrosiers , M. Cornet , et al., “Efficacy and Safety of Twice per Year Depemokimab in CRSwNP (ANCHOR‐1 and ANCHOR‐2): Phase 3, Randomised, Double‐Blind, Parallel Trials,” The Lancet 405, no. 10482 (2025): 911–926. 10.1016/S0140-6736(25)00197-7 40037388

[12] C. Bachert , J. K. Han , M. Desrosiers , et al., “Efficacy and Safety of Dupilumab in Patients With Severe CRSwNP (LIBERTY NP SINUS‐24 and LIBERTY NP SINUS‐52): Results From Two Multicentre, Randomised, Double‐Blind, Placebo‐Controlled, Parallel‐Group Phase 3 Trials,” The Lancet 394, no. 10209 (2019): 1638–1650. 10.1016/S0140-6736(19)31881-1 31543428

[13] H. Li , C. Wang , and C. Guo , “A Pharmacovigilance Analysis of Post‐Marketing Safety of Tezepelumab,” Journal of Allergy and Clinical Immunology: In Practice 13, no. 3 (2025): 551–558.e6, 10.1016/j.jaip.2024.10.045. 39521341

[14] Z. Mao , Y. Huang , X. Zhu , et al., “Adverse Events Associated With Tezepelumab: A Safety Analysis of Clinical Trials and a Pharmacovigilance System,” Expert Opinion on Drug Safety 25 (2024): 291–300, 10.1080/14740338.2024.2416921. 39422097

